# Supplementary figures and images for: Spatial layout optimization model integrating layered attention mechanism in the development of smart tourism management
Source: PeerJ Comput Sci. 2024 Oct 9;10:e2329. doi: 10.7717/peerj-cs.2329 (PMC11623231; doi:10.7717/peerj-cs.2329)

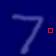

Supplement: Supplemental Information 5 [file peerj-cs-10-2329-s005.zip › 重新上传代码-code/AttentionModule/Non-local/nl_map_vis/nl_map_1/110.png]

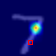

Supplement: Supplemental Information 5 [file peerj-cs-10-2329-s005.zip › 重新上传代码-code/AttentionModule/Non-local/nl_map_vis/nl_map_1/147.png]

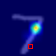

Supplement: Supplemental Information 5 [file peerj-cs-10-2329-s005.zip › 重新上传代码-code/AttentionModule/Non-local/nl_map_vis/nl_map_1/161.png]

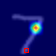

Supplement: Supplemental Information 5 [file peerj-cs-10-2329-s005.zip › 重新上传代码-code/AttentionModule/Non-local/nl_map_vis/nl_map_1/174.png]

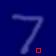

Supplement: Supplemental Information 5 [file peerj-cs-10-2329-s005.zip › 重新上传代码-code/AttentionModule/Non-local/nl_map_vis/nl_map_1/177.png]

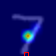

Supplement: Supplemental Information 5 [file peerj-cs-10-2329-s005.zip › 重新上传代码-code/AttentionModule/Non-local/nl_map_vis/nl_map_1/188.png]

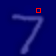

Supplement: Supplemental Information 5 [file peerj-cs-10-2329-s005.zip › 重新上传代码-code/AttentionModule/Non-local/nl_map_vis/nl_map_1/37.png]

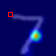

Supplement: Supplemental Information 5 [file peerj-cs-10-2329-s005.zip › 重新上传代码-code/AttentionModule/Non-local/nl_map_vis/nl_map_1/44.png]

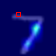

Supplement: Supplemental Information 5 [file peerj-cs-10-2329-s005.zip › 重新上传代码-code/AttentionModule/Non-local/nl_map_vis/nl_map_1/46.png]

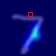

Supplement: Supplemental Information 5 [file peerj-cs-10-2329-s005.zip › 重新上传代码-code/AttentionModule/Non-local/nl_map_vis/nl_map_1/49.png]

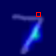

Supplement: Supplemental Information 5 [file peerj-cs-10-2329-s005.zip › 重新上传代码-code/AttentionModule/Non-local/nl_map_vis/nl_map_1/51.png]

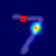

Supplement: Supplemental Information 5 [file peerj-cs-10-2329-s005.zip › 重新上传代码-code/AttentionModule/Non-local/nl_map_vis/nl_map_1/61.png]

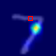

Supplement: Supplemental Information 5 [file peerj-cs-10-2329-s005.zip › 重新上传代码-code/AttentionModule/Non-local/nl_map_vis/nl_map_1/63.png]

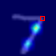

Supplement: Supplemental Information 5 [file peerj-cs-10-2329-s005.zip › 重新上传代码-code/AttentionModule/Non-local/nl_map_vis/nl_map_1/66.png]

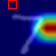

Supplement: Supplemental Information 5 [file peerj-cs-10-2329-s005.zip › 重新上传代码-code/AttentionModule/Non-local/nl_map_vis/nl_map_2/1.png]

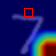

Supplement: Supplemental Information 5 [file peerj-cs-10-2329-s005.zip › 重新上传代码-code/AttentionModule/Non-local/nl_map_vis/nl_map_2/10.png]

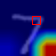

Supplement: Supplemental Information 5 [file peerj-cs-10-2329-s005.zip › 重新上传代码-code/AttentionModule/Non-local/nl_map_vis/nl_map_2/18.png]

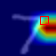

Supplement: Supplemental Information 5 [file peerj-cs-10-2329-s005.zip › 重新上传代码-code/AttentionModule/Non-local/nl_map_vis/nl_map_2/19.png]

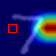

Supplement: Supplemental Information 5 [file peerj-cs-10-2329-s005.zip › 重新上传代码-code/AttentionModule/Non-local/nl_map_vis/nl_map_2/22.png]

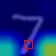

Supplement: Supplemental Information 5 [file peerj-cs-10-2329-s005.zip › 重新上传代码-code/AttentionModule/Non-local/nl_map_vis/nl_map_2/38.png]

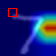

Supplement: Supplemental Information 5 [file peerj-cs-10-2329-s005.zip › 重新上传代码-code/AttentionModule/Non-local/nl_map_vis/nl_map_2/8.png]

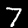

Supplement: Supplemental Information 5 [file peerj-cs-10-2329-s005.zip › 重新上传代码-code/AttentionModule/Non-local/nl_map_vis/sample.png]

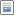

Supplement: Supplemental Information 5 [file peerj-cs-10-2329-s005.zip › 重新上传代码-code/sphinx/build/html/_static/file.png]

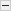

Supplement: Supplemental Information 5 [file peerj-cs-10-2329-s005.zip › 重新上传代码-code/sphinx/build/html/_static/minus.png]

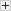

Supplement: Supplemental Information 5 [file peerj-cs-10-2329-s005.zip › 重新上传代码-code/sphinx/build/html/_static/plus.png]
